# The Modulation of Phosphatase Expression Impacts the Proliferation Efficiency of HSV-1 in Infected Astrocytes

**DOI:** 10.1371/journal.pone.0079648

**Published:** 2013-11-15

**Authors:** Lei Yue, Sujie Guo, Ying Zhang, Longding Liu, Qingling Wang, Xi Wang, Dong Shen, Lichun Wang, Le Sun, Jingjing Wang, Yun Liao, Qihan Li

**Affiliations:** Institute of Medical Biology, Chinese Academy of Medicine Science & Peking Union Medical College, Kunming, People’s Republic of China; University of Pennsylvania School of Veterinary Medicine, United States of America

## Abstract

Herpes Simplex Virus 1 (HSV-1) is a major pathogen that causes human neurological diseases, including herpes simplex encephalitis (HSE). Previous studies have shown that astrocytes are involved in HSV-1 systemic pathogenesis in the central nervous system (CNS), although the mechanism remains unclear. In this study, a high-throughput RNAi library screening method was used to analyze the effect of host phosphatase gene regulation on HSV-1 replication using *Macaca mulatta* primary astrocytes in an *in vitro* culture system. The results showed that the downregulation of five phosphatase genes (PNKP, SNAP23, PTPRU, LOC714621 and PPM1M) significantly inhibited HSV-1 infection, suggesting that these phosphatases were needed in HSV-1 replication in rhesus astrocytes. Although statistically significant, the effect of downregulation of these phosphatases on HSV-1 replication in a human astrocytoma cell line appears to be more limited. Our results suggest that the phosphatase genes in astrocytes may regulate the immunological and pathological reactions caused by HSV-1 CNS infection through the regulation of HSV-1 replication or of multiple signal transduction pathways.

## Introduction

Herpes Simplex Virus 1 (HSV-1), a DNA virus with a complex gene expression program [1], can not only manipulate transcription regulation leading to latent infection [2], but also cause comprehensive pathological injury, such as herpes simplex encephalitis (HSE) with poor prognosis, in hosts during its infection process [3], [4]. The studies of the mechanism of HSV-1 virus genetic transcription regulation and HSV-1 pathogenesis have shown that the interaction between HSV-1 and the host largely determines the specific virus infection process [5], [6]. Therefore, studies of the molecular events of interactions between viruses and host cells may provide direct evidence for understanding the mechanism of HSV-1 pathogenesis.

Previous studies have shown that astrocytes in the central nervous system (CNS) are involved in the pathogenesis of HSV-1 encephalitis [7]. This involvement occurs because astrocytes, with a fast response to invasive pathogens [8], are important immune cells in the CNS, and because of the capability of HSV-1 to infect astrocytes and promote systemic pathogenesis in the CNS [9]. Studies have suggested that production of certain inflammatory factors, such as MCP-1 and TNF, were increased following HSV-1 infection in astrocytes [10], which was observed to be mediated in a TLR-2-dependent manner [10]. Although the mechanism of interaction between host and HSV-1 in astrocytes, especially the impact of cytokine production on the viral replication, is not clear, the phosphatases involved in the TLR2 response signaling pathway were confirmed to be very important components [11], [12]. Thus, to explore the role of certain phosphatases in HSV-1 replication might be helpful to yield insight into the cellular response to viral infection and into their further significance in the pathogenesis of HSV-1 infection in astrocytes, which is a part of the pathological process of encephalitis induced by this virus [13]. Therefore, our work in this study was focused on the interaction of phosphatases with HSV-1 in astrocytes to better understand the molelular mechanism HSV-1 pathogenesis in infectious encephalitis.

Studies of the biological activities of astrocytes have shown that as a major component of the CNS, astrocytes play important roles in neuron regulation and immune responses [14], [15]. In general, the neuron-astrocyte communication is very important for the systemic stability of the CNS [16], which includes both the activation of astrocytes by synaptically released neurotransmitters and their signaling back to neurons [16]. During HSV-1 infection, upregulation of cytokine release by infected astrocytes may alter the normal neuron-astrocyte communication and lead to local instability of the CNS. [17]. This study investigated the mechanism involved in HSV-1 infection of astrocytes. We focused on using an RNAi library screening method to evaluate the role of phosphatases on HSV-1 infection of astrocytes. We targeted phosphatases directly involved in cell regulation to understand the molecular mechanism of the change in the biological activities of astrocytes during HSV-1 infection.

## Materials and Methods

### Ethics statement

All animal experimental procedures were carried out in strict accordance with the National Institute of Health Guidelines for the Care and Use of Laboratory Animals. The experimental animal procedure was approved by the Office of Laboratory Animal Management of Yunnan Province and the Ethics Committee of the Institute of Medical Biology. The monkey was kept and bred according to the guidelines of the committee of experimental animals at the Institute of Medical Biology, Chinese Academy of Medical Sciences (CAMS) [18].

We used a single monkey, with a weight of 3 kg and an age of 3.5 years, for each primary culture of astrocytes; astrocyte collection was performed three times in this study. Before the initiation of the study, the monkey was kept in isolation for 1 day from a group that is provided with toys and facilities to allow swinging, jumping and engaging in activities from floor to ceiling. The monkey was housed in a pen with a height of 1.8 m and an area of 2.5 m^2^ in a light-dark (LD) 12:12 cycle (12 hours light, 12 hours dark). This monkey has constant access to water which had been purified with filters, sterilized or additionally chlorinated or acidified in order to eliminate biological pollution and was fed every day with sterilized foods and fresh apples.

This monkey was euthanized with sodium pentobarbital (10 mg/kg). The cerebral material was obtained 15 minutes after the sacrifice of the animal.

### Preparation of monkey cerebrocortical astrocytes

Astrocytes were prepared from the cortex of adult monkeys using a protocol derived from previously described methods [19]–[21]. Cortices were washed several times with PBS, cut into small cubes (<1 mm^3^) and digested with 0.25% trypsin for 25 min at 37°C. Trypsinization was terminated by the addition of Dulbecco’s modified Eagle’s medium (DMEM) that contained 10% fetal bovine serum (FBS) (GIBCO). Cell suspensions were sieved through a 40 mm cell strainer. The filtrate was seeded at a density of 1×10^6^ cells/cm^2^ in 100 mm dishes (Corning, USA) after being pre-adhered for 30 min to remove fibroblasts. The plated cells were cultured in a 5% CO_2_ incubator at 37°C for 21 days and the culture medium was changed at 5-day intervals as described [22], [23]; After confluence, the primary cell culture was washed 3 times with PBS and digested with 0.25% trypsin at room temperature for 5 min. Trypsinization was also terminated by the addition of DMEM containing 10% fetal bovine serum. After centrifugation, the cells were subsequently cultured in DMEM containing 10% fetal calf serum, 100 U/ml penicillin and 20 µg/ml streptomycin in a 5% CO_2_ incubator at 37°C. Other glia, such as oligodendroglia and microglia were removed during this procedure. The purity of astrocytes was assessed by the percentage of cells showing fluorescence for the glial fibrillary acidic protein (GFAP) marker.

### SiRNA transfection

Both the siRNA used for knockdown and the siRNA used as a control were designed and synthesized by Guangzhou RiboBio Co., Ltd. One day before transfection, the cells were plated in growth medium without antibiotics. The cell monolayer was then transfected with siRNA at a final concentration of 50 nM with the Lipofectamine™ 2000 transfection reagent (Invitrogen) according to the manufacturer’s instructions.

### Cell lines and Viruses

HEK293 and Vero cells were grown in Modified Eagle Medium (MEM) supplemented with 10% newborn bovine serum (GIBCO). U-87MG cells were grown in Dulbecco's Modified Eagle's Medium (DMEM) supplemented with 10% fatal bovine serum (GIBCO). Stocks of HSV-1(strain 17) [5] were prepared in Vero cells and gradient purified.

### Growth curve analysis

The cell monolayers were then infected with HSV-1 at a MOI of 0.01 PFU/cell. The cell culture supernatants were harvested at 0, 12, 24, 36, 48, 60, 72, 84 and 96 hr post-infection., clarified and stored at –80°C. HSV-1 titers were determined by a plaque assay in Vero cells. Monolayers were infected with HSV-1 and overlaid with 700 µL essential medium containing 2% FBS in 6-well plates. After virus adsorption, monolayers were overlaid with 2.5 mL essential medium containing 2% FBS and 0.9% agarose. After fixation of the cells with 4% formaldehyde (Solarbio) and removal of the agarose overlay at 4–5 days post-infection, the plaques were visualized by staining with 1% crystal violet (Solarbio) in distilled water.

### Immunofluorescence

Primary cell cultures on coverslips were washed with PBS, fixed in 4% paraformaldehyde and permeabilized with 0.2% Triton X-100. The cells were incubated with a rabbit anti-GFAP antibody (1∶8) for 1 hr and sebsequently incubated with a FITC-conjugated goat anti-rabbit antibody (1∶250) for 1 hr. The slides were examined using a Nikon E600 fluorescence microscope.

### SiRNA library screening

All 212 monkey phosphatase genes and putative phosphatase genes were chosen from the NCBI. The siRNAs (3 per gene) targeting the phosphatases were designed and purchased from Guangzhou RiboBio Co., Ltd. Three siRNAs for each gene target were cotransfected into monkey astrocytes for screening in 96-well plates.

Forty-eight hours after transfection (described above), the cells were infected with HSV-1 (MOI = 0.01). Forty-eight hours after infection, the titer of each well was tested by microtitration assay. Briefly, viral stocks were serially diluted 10 times, and 100 µL of the diluted stocks were added to Vero cells with 80% confluence in each of the 96-well plates. Overall, 8 parallel wells for each dilution stock were used. The 96-well plates were incubated at 37°C in a 5% CO_2_ incubator for 5–7 days to observe the cytopathic effects. The 50% cell culture infective dose (CCID50) was determined by Reed-Muench assay.

### Real-time PCR

Total RNA was extracted from the transfected astrocyte cells and reverse-transcribed to cDNA using the RevertAid First Strand cDNA Synthesis kit (Fermentas). Real-time PCR was performed in the 7500 Fast Real-time RT-PCR system (Applied Biosystems) using the SYBR Green RealMaster kit (Tiangen) and the primers 5′- ACC ATT CCC ACG TCT TCA CCT TTG-3′ and 5′- AGA CAT TCT CTC GTT CAC CGC C-3′for *ALPL*; 5′-TCA TCC AAC GGC TGC TGG AA-3′ and 5′- TGA TGC TGG CAC ATT CAT GG-3′for *PPP1CC*; 5′-ATC TCC ATC CGG GAC AGC ATC TTT-3′ and 5′-CAA GGT TGA GGG CAA ACA AGC GAT-3′ for *PNKP*; 5′-GAC CCA TCA TCA TCC ACA C-3′ and 5′-CAT CCC TTC CTT CAC CTT TT-3′ for *ACP6*; 5′-TCC ACA CCC AGA CCC AAA A-3′ and 5′-GGG CTT CAC ATT CCA AGA G-3′ for *DUSP11*; 5′-TTG GTA CCA TGG ATA TGA CAT CGC GGA GAT GG-3′ and 5′-AGC TCG AGT CAT CTG AAA CTT TTC TGC TGT T-3′ for *PTPN11*; 5′-GGC ATC CTC ACC CTG AAG TA--3′ and 5′-GGG GTG TTG AAG GTC TCA AA-3′ *for β-actin* (normalization control). The following protocol was used for these PCR assays: 3 min at 95°C, followed by 40 cycles of 95°C for 10 s, 56°C for 10 s and 68°C for 40 s. All experiments were repeated three times.

### Electron microscopy analysis

The tissue was prepared and fixed in a solution containing 0.5% glutaraldehyde, 4% paraformaldehyde and 0.1 M of sodium phosphate buffer (pH 7.6) on ice for 1 hr. After washes with 4% sucrose in 0.1 M phosphate buffer, the tissue was decalcified with a 2.5% EDTA solution for 5 days. After three washes, the samples were fixed with 2% osmium tetraoxide on ice for 1 hr and dehydrated with a series of ethanol gradients. For transmission electron microscopy, the tissues were embedded in Epon 812 resin mixture, and ultrathin (70-nm) sections were cut with Leica EM UC6 ultramicrotome (Leica Co., Austria). The ultrathin sections were stained with 2% uranyl acetate in 70% ethanol for 5 min at room temperature and then with Reynold's lead for 5 min at room temperature. Sections were analyzed with an H-7650 transmission electron microscope (HITACHI, Japan).

### Statistical analysis

The statistical analysis was performed using SPSS 13.0 software. Data obtained from all experiments performed in triplicate were processed by variance analysis, and *P*<0.05 was considered to be statistically significant.

## Results

### Replication of HSV-1 in Macaca mulatta astrocytes

**Figure 1:**
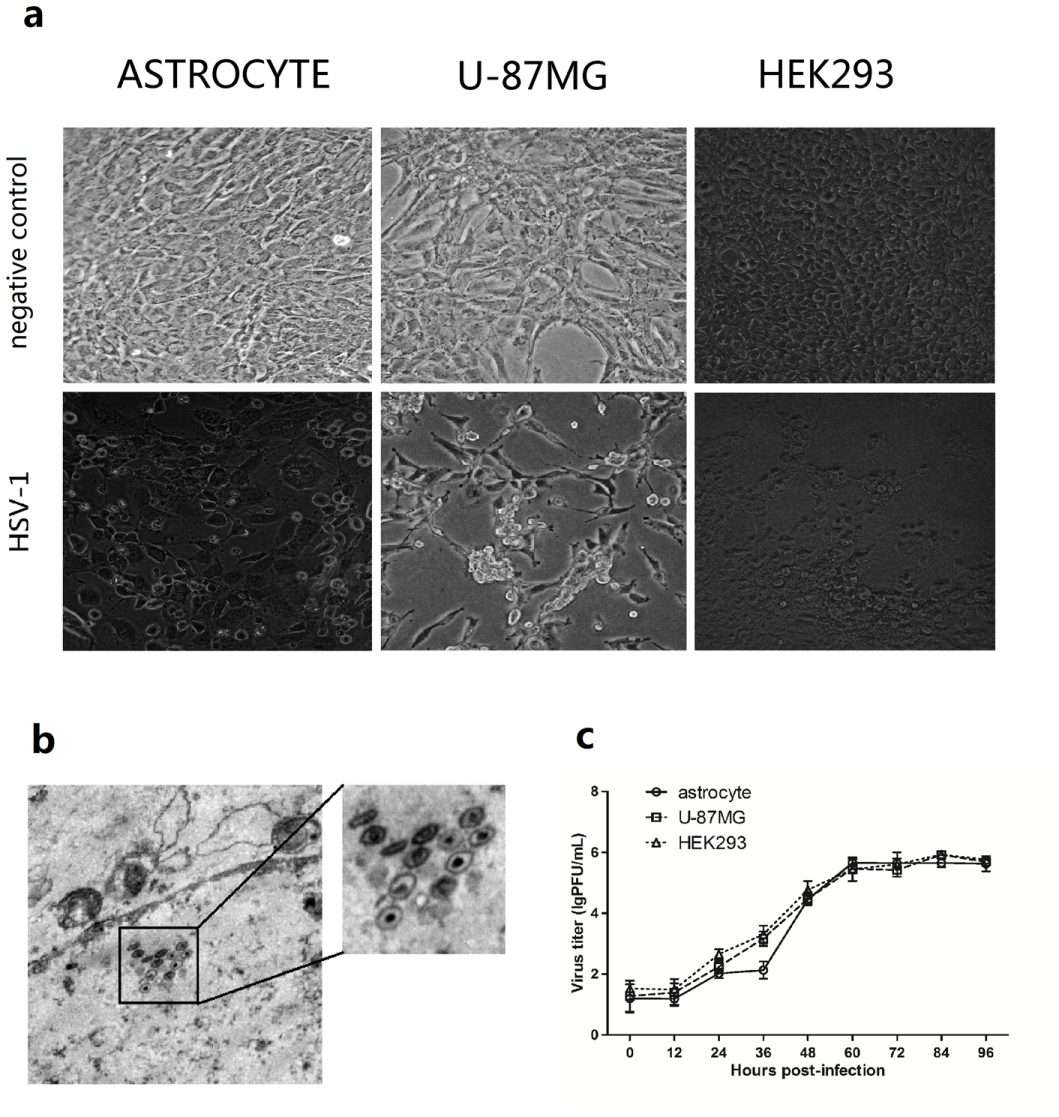
Replication of HSV-1 in *Macaca mulatta* astrocytes. (a) HSV-1 infection of primary astrocytes. Cellular morphology of astrocyte, U-87MG and HEK293 before and after HSV-1 infection under the light microscope; Upper panels: negative control; Lower panels: 12 hours post HSV-1 infection (MOI = 1). The magnification is 200 X. (b) Virus morphology in astrocytes infected with HSV-1. The monolayer of astrocytes was infected with HSV-1 (MOI  = 2). The infected astrocytes were observed at 24 h post infection using Transmission Electron Microscope (TEM), and the typical structure was observed for HSV-1 virus capsids. The magnification is 6000 X. (c) Viral replication curve in rhesus primary astrocytes, U-87MG and HEK293 cells infected with HSV-1. The primary astrocytes, U-87MG and HEK293 cells were infected with HSV-1 (MOI  = 0.01). Samples were collected at 0, 12, 24, 36, 48, 60, 72, 84 and 96 hr post infection. A plaque assay was used to determine the virus titer in all of the samples to generate the viral replication curve. Error bars represent the standard deviation from triplicate samples.

The pathological study of HSE caused by HSV-1 suggested that replication in the CNS was one of the major events in HSV-1 pathogenesis [4]. Although studies have confirmed that HSV-1 can cause latent infections in neurons [24], the HSV-1 replication mechanism in neurons is still not clear due to the special biological environment of neurons. Therefore, astrocytes, which are important cell components in the CNS, are one of the major target cells study for a better understanding of HSV-1 in encephalitis pathogenesis. An astrocyte tissue culture system (purity > 90%) was obtained using a differential adsorption method of passaging of astrocytes from a *M. mulatta* CNS. The purity of the cell culture was verified using fluorescence detection with a specific antibody against the glial fibrillary acidic protein (GFAP), which is expressed in astrocytes [Fig.S1]. HSV-1 (MOI  =  0.01) was used to infect astrocytes in the astrocyte culture system, and virus replication was observed. As the control, human astrocytoma cells U-87MG strain, and HEK293 cells were also used. The results showed that after HSV-1 infection, astrocytes showed obvious cytopathic effects (CPE), similar to the effects of HSV-1 infection of U-87MG and HEK293 cells [Fig.1a]. In addition, transmission electron microscope (TEM) results confirmed the microscopic cellular structure change and the virus replication in rhesus astrocytes [Fig.1b]. A subsequent kinetics study involving HSV-1-infected astrocytes at different time points post-infection showed that the infection had typical viral infection kinetics [25] [Fig.1c]. The HSV-1 replication kinetics in astrocytes were similar to the replication kinetics the U-87MG and HEK293 cell lines.

### Phosphatase siRNA library transfection of astrocytes

**Figure 2:**
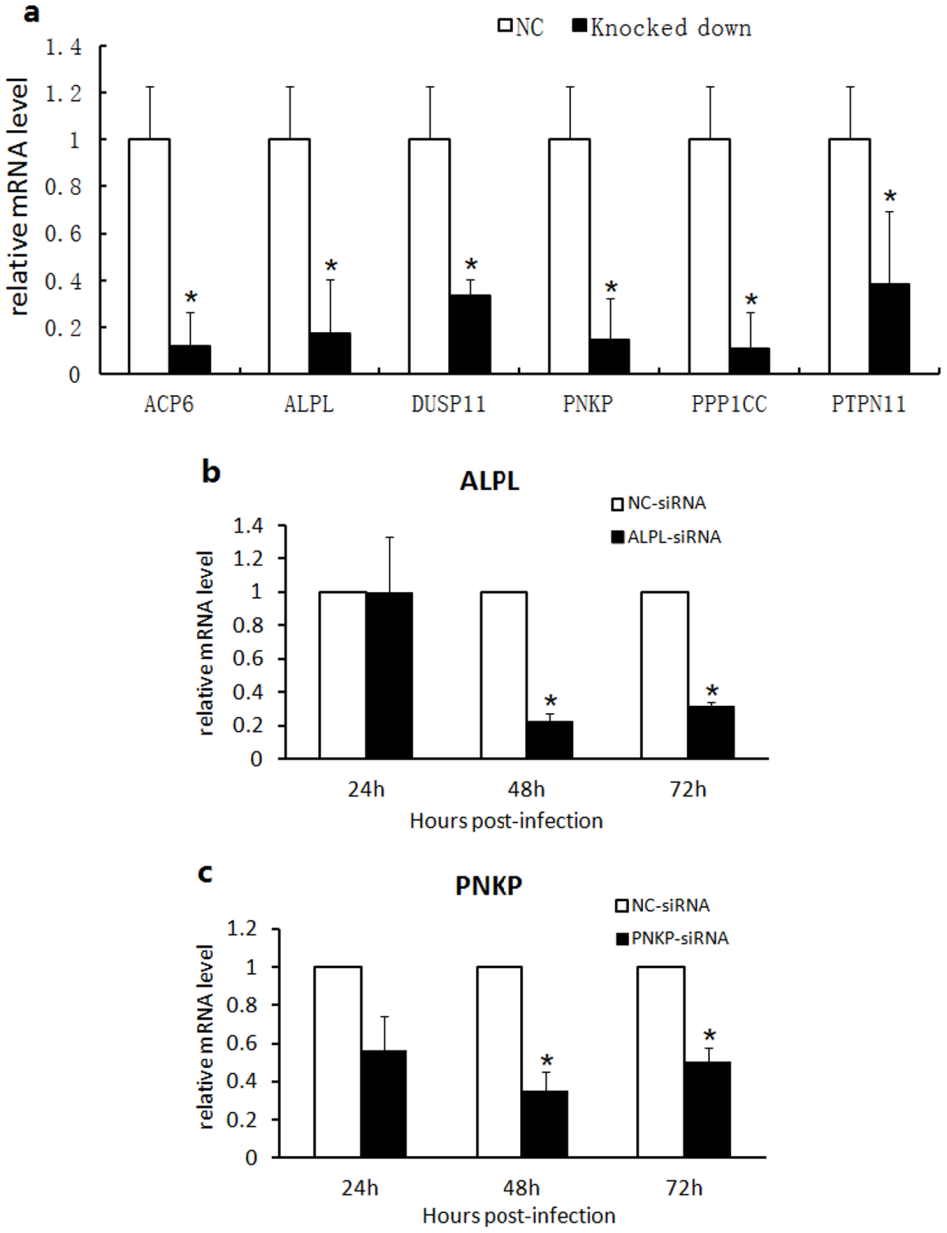
siRNA transfection effectively downregulated corresponding phosphatase RNA expression in astrocytes. (a) Determination of the silencing effect of six genes: APP6, ALPL, DUSP11, PPP1CC, PTPN11 and PNKP. The siRNAs of the six genes (ACP6, ALPL, DUSP11, PPP1CC, PTPN11 and PNKP), were used to transfect astrocytes (50 nM). Total RNA was extracted at 48 h post transfection; a relative quantification method was used to calculate the target gene transcription. Data were analyzed using 2^−ΔΔCt^. β-actin was used as the internal control. Error bars represent the standard deviation from triplicate samples. * indicates P<0.05. (b,c) Relationship between transfection time and the silencing effect of the ALPL and PNKP genes. The siRNAs of the ALPL and PNKP genes were used to transfect astrocytes (50 nM), and the total RNA was extracted at 24, 48 and 72 hr post transfection; a relative quantification method was used to calculate the target gene transcription. Data were analyzed using 2^−ΔΔCt^. β-actin was used as the internal control. Error bars represent the standard deviation from triplicate samples. * indicates P<0.05.

Astrocytes have multiple regulatory functions in the CNS [17]. We designed the siRNA library for phosphatase systems [26] involved in cellular activity in astrocytes. This library included siRNAs for 212 phosphatase genes [Table S1] and was used to screen phosphatases involved in HSV-1 infection as well as to further understand the molecular mechanism of the interaction between HSV-1 and astrocytes. Our study first investigated the efficiency of the siRNA transfection in astrocytes. Cyanine 3 (Cy3)-labeled siRNA transfection results showed that the transfection method was successful [Fig.S2]. Six phosphatase genes (ACP6, ALPL, DUSP11, PNKP, PPP1CC and PTPN11) were further analyzed using qRT-PCR, and the results showed that siRNA transfection effectively downregulated corresponding phosphatase RNA expression [Fig. 2a]. The downregulation showed a three- to fivefold times reduction compared with the control RNA expression. Two phosphatase genes (ALPL and PNKP) were used to study the inhibitory kinetics, and the results showed that the inhibition was effective from 24 to 72 hr post-transfection [Fig. 2b,c]. All of these results confirmed the effectiveness of the phosphatase siRNA transfection-inhibition system.

### The effect of the downregulation of 212 phosphatase genes in astrocytes on HSV-1 replication

**Figure 3:**
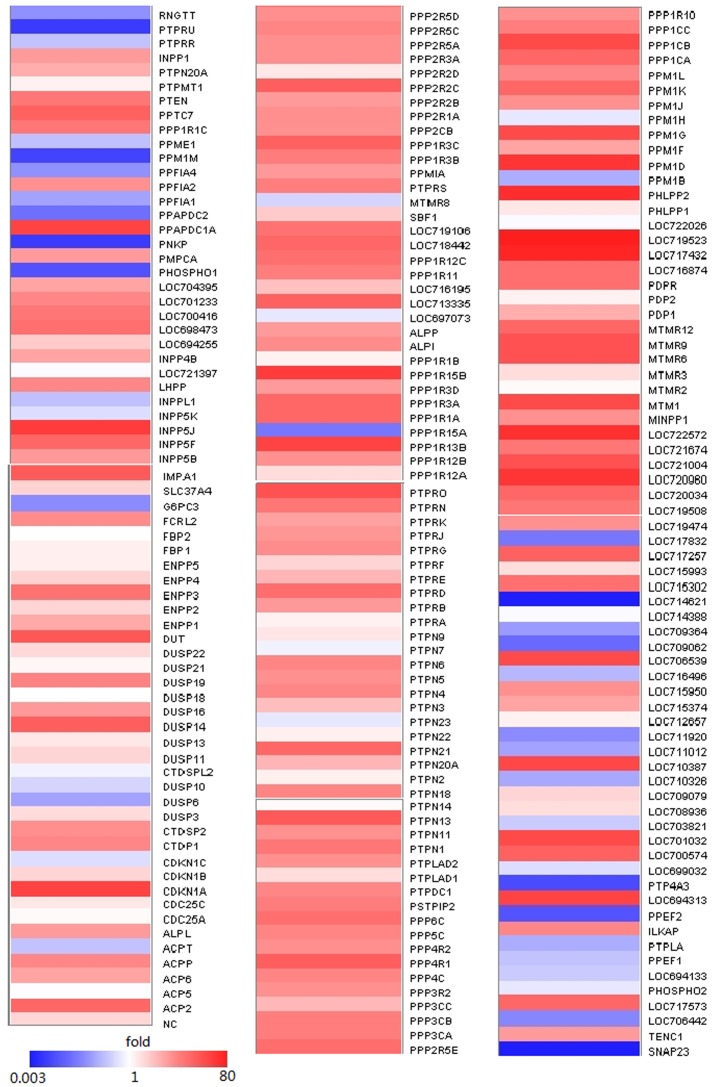
siRNA knockdown of different phosphatases led to different responses to HSV-1 infections. Three siRNAs per gene were transfected in astrocytes followed by infection with HSV-1 (at an MOI of 0.01) in three independent experiments. Virus solutions were harvested at 48 h post infection, and the virus titer was determined by the CPE method and analyzed by calculating the normalized fold of CCID50/mL. The color scale reflects the fold of reduction or increase level of viral replication. These values (0.003-80) represent ratios of CCID50/mL (target/NC). NC refers to the negative control (value considered as 1).

The siRNA for each phosphatase was used to transfect astrocytes (3 siRNAs per gene, 3 replicate wells per gene). HSV-1 (MOI  = 0.01) was used to infect astrocytes at 48 hr post- transfection. Viruses were harvested at 48 hr post-infection, and the titer was determined. Non-functional siRNA (NC-siRNA) transfection of astrocytes was used as the negative control. The results showed that with siRNA knockdown of different phosphatases led to different cellular responses to HSV-1 infections [Fig.3]. However, siRNA knockdown of most phosphatases did not affect virus replications significantly; only the knockdown of five phosphatases led to significant decreases (>100x reduction of HSV-1 replication) in virus infection efficiency [Fig.S3 and Table S2].

### The effect of five phosphatases on HSV-1 replication efficiency in astrocytes

**Figure 4:**
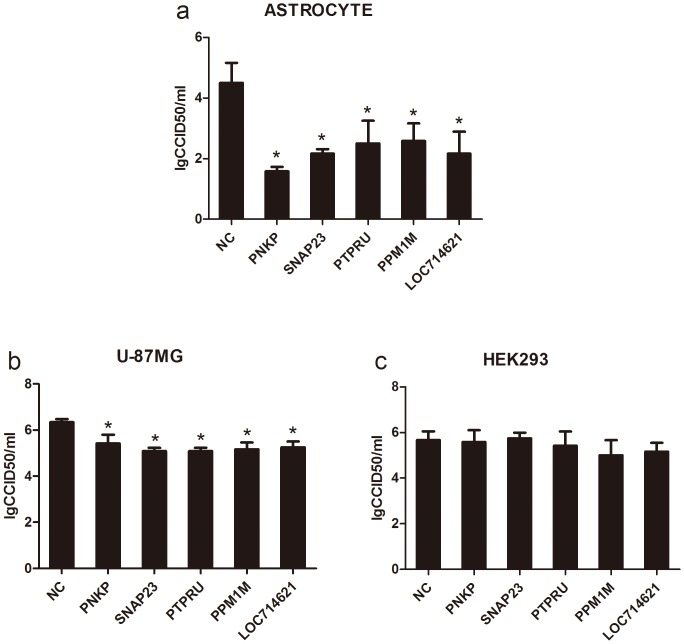
PNKP, SNAP23, PTPRU, LOC714621 and PPM1M genes in astrocytes were identified as key genes affecting HSV-1 replication. Three siRNAs per gene (PNKP, SNAP23, PTPRU, LOC714621 and PPM1M) were transfected in astrocytes (a), U-87MG (b) and HEK293 (c) followed by infection with HSV-1 (at an MOI of 0.01) in three independent experiments. Virus solutions were harvested at 48 h post infection, and the virus titer was determined by the CPE method. * indicates P<0.05.

The study of HSV-1 infection efficiency with the downregulation of 212 phosphatase genes showed that the downregulation of five phosphatases (PNKP, SNAP23, PTPRU, LOC714621 and PPM1M) by siRNA transfection led to significant decreases in the HSV-1 titer [Fig. 4a]. The downregulation of these phosphatases can cause a 100- to 1000-fold reduction in HSV-1 infection. This result suggested the possibility that these five phosphatases were needed for the replication of HSV-1 in astrocytes and that these phosphatases, which are involved in cellular signal transduction, might contribute to the pathogenesis of HSV-1 encephalitis by mediating cytokine upregulation. Although the complete data regarding these phosphatases are not yet available, the fact that these five phosphatases were observed to be required in human astrocytes [Fig. 4b] but not HEK293 cells [Fig. 4c] seems supportive of this possibility. The downregulation of these five phosphatases can cause a 5- to 20-fold reduction in HSV-1 infection in U-87MG [Fig. 4b]. Thease results indicated that the effect of downregulation of these phosphatases on HSV-1 replication in a human astrocytoma cell line appears to be more limited. Additionally, further study of the functions of these five phosphatases suggested that the phosphatases were involved in the phosphorylation regulation of several functional molecules and structural proteins in cells [Table 1].

**Table 1:** Identification of Phosphatase Genes Associated with HSV-1 Replication.

Accession	Symbol	Product	Interaction
XM_001115581.1	PNKP	polynucleotide kinase 3'-phosphatase	XRCC4, LIG3, LigIV
XM_002804738.1	SNAP23	synaptosomal-associated protein 23-like	synaptobrevin/VAMP
XM_001115805.2	PTPRU	protein tyrosine phosphatase, receptor type, U	CTNNB1
XM_001104480.2	LOC714621	myotubularin-related protein 4-like	SMAD3, UBC
NM_001193781.1	PPM1M	protein phosphatase, Mg2+/Mn2+ dependent, 1M	unknown

## Discussion

HSV-1, which is a common virus found in human populations [27], has been proven to cause numerous diseases through complicated pathogenesis pathways. Among all of these diseases, HSE has a low occurrence but a poor prognosis [28], as well as important clinical significance. The mechanism by which HSV-1 causes HSE remains unclear. However, a previous study of HSV-1 infection in astrocytes indicated that the infected astrocytes experienced an upregulation of various cytokines which caused instability of neuron-astrocyte communication and subsequently the dysfunction of the CNS [8]. Therefore, analysis of the interaction between HSV-1 and astrocytes, which are important functional cells in the CNS, may provide important evidence for understanding the clinical pathogenesis of HSE. This study investigated the interaction between HSV-1 and cellular phosphatases involved in the regulation of HSV-1 infection using siRNA interference of 212 phosphatases in astrocytes to observe the titer changes in HSV-1. The results showed that the downregulation of five phosphatases had significant inhibitory effects on HSV-1 infection, suggesting that these phosphatases were needed for HSV-1 replication in astrocytes. This observation was extended to human astrocytoma cells, in which the downregulation of these five phosphatases inhibited the proliferation of HSV-1 and implied a role of these five phosphatases in the replication of HSV-1 in astrocytes. Studies have shown that among the five phosphatases (PNKP, SNAP23, PTPRU, LOC714621 and PPM1M ), the PNKP-relevant molecules XRCC4 and LigIV have been demonstrated to play a role in the circularization of HSV-1 linear double-stranded genomes [29]. In addition, PNKP has been shown to be involved in double-strand break (DSB) repair [30] with its 5′-DNA kinase and 3′-DNA phosphatase activities [31]. These functions which are directly involved in HSV-1 genome replication indicated that these phosphatases have important roles in HSV-1 replication in astrocytes. The regulatory molecules (SNAP23, PTPRU and LOC714621) involved in different cellular signal transduction pathways showed an inhibitory effect on HSV-1 replication efficiency, suggesting that these signaling pathways can directly or indirectly affect the HSV-1 replication mechanism [32]–[34]. While there is data to suggest that SNAP23 is the components of the TLR signaling pathway [35], our findings suggested the immunological response of astrocytes may influence HSV-1 replication. Clearly, while more exploration is needed to understand the details of the mechanism of this process, this study has provided insight for further studies regarding not only the interaction of HSV-1 and astrocytes but also the pathogenesis of encephalitis induced by this virus.

## Supporting Information

Figure S1 **Indirect immunofluorescence staining for GFAP in primary astrocytes.** The magnification is 200 X.
(TIF)

Figure S2 **Effective transfection of astrocytes with siRNA.** Cy-3 labeled NC-siRNA (50 nM) was used to transfect astrocyte monolayers. The transfection efficiency was calculated using fluorescence microscope at 48 h post-infection. The magnification is 200 X.
(TIF)

Figure S3 **Virus titer for each phosphatase in monkey astrocytes by siRNA screen.** Three siRNAs per gene were transfected in astrocytes followed by infection with HSV-1 (at an MOI of 0.01) in three independent experiments. Virus solutions were harvested at 48 h post infection, and the virus titer was determined by the CPE method and analyzed by calculating the lgCCID50/mL. Cut off = 2.7, and it means 100x reduction of HSV-1 replication. NC refers to the negative control.
(TIF)

Table S1 **List of phosphatases in the siRNA library.**
(DOCX)

Table S2 **Normalized virus titers for RNAi screening.**
(DOCX)
