# Supplementary figures and images for: The Modulation of Phosphatase Expression Impacts the Proliferation Efficiency of HSV-1 in Infected Astrocytes
Source: PLoS One. 2013 Nov 15;8(11):e79648. doi: 10.1371/journal.pone.0079648 (PMC3829861; doi:10.1371/journal.pone.0079648)

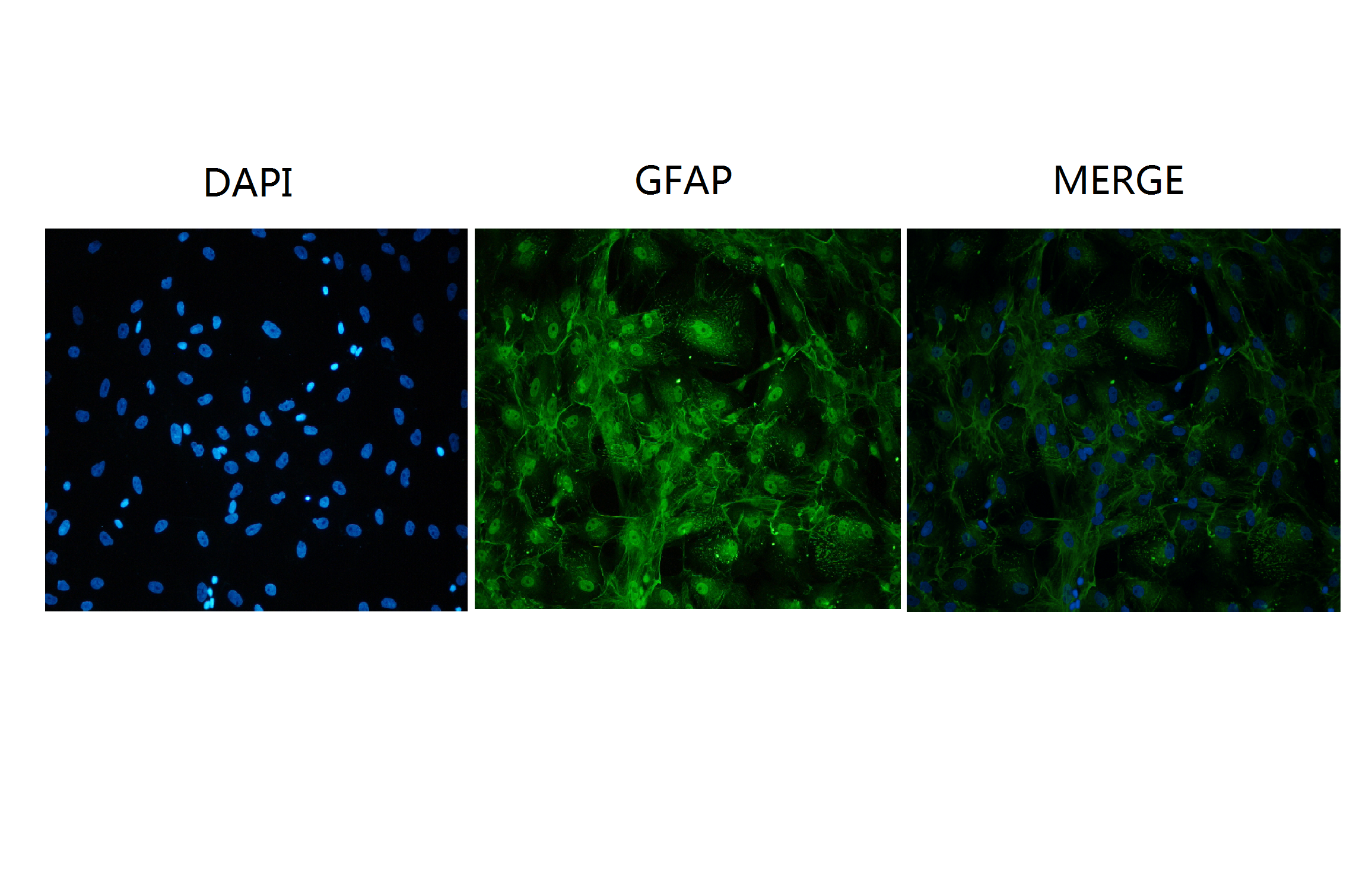

Supplement: Figure S1 — Indirect immunofluorescence staining for GFAP in primary astrocytes. The magnification is 200 X. (TIF) [file pone.0079648.s001.tif]

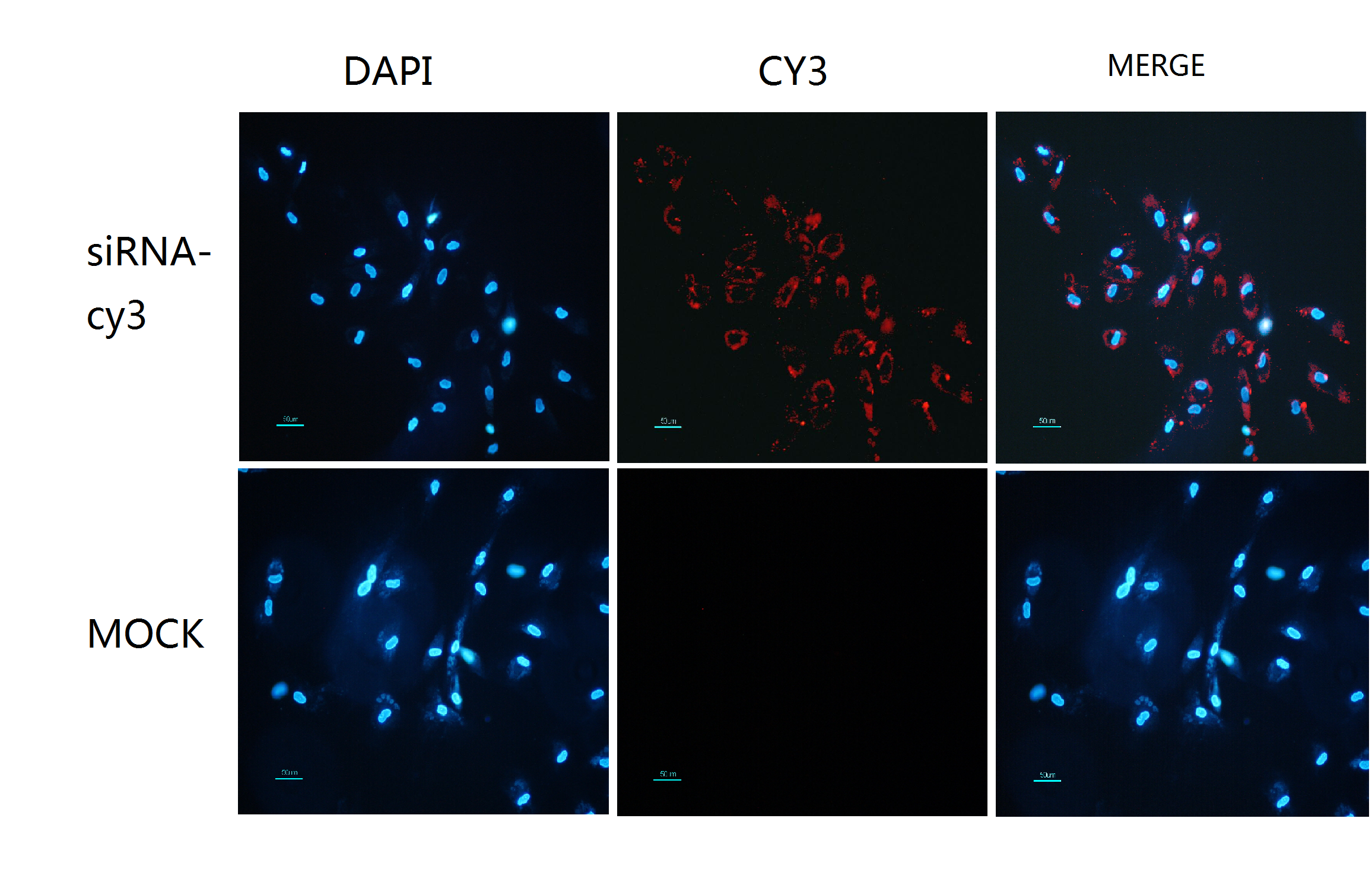

Supplement: Figure S2 — Effective transfection of astrocytes with siRNA. Cy-3 labeled NC-siRNA (50 nM) was used to transfect astrocyte monolayers. The transfection efficiency was calculated using fluorescence microscope at 48 h post-infection. The magnification is 200 X. (TIF) [file pone.0079648.s002.tif]

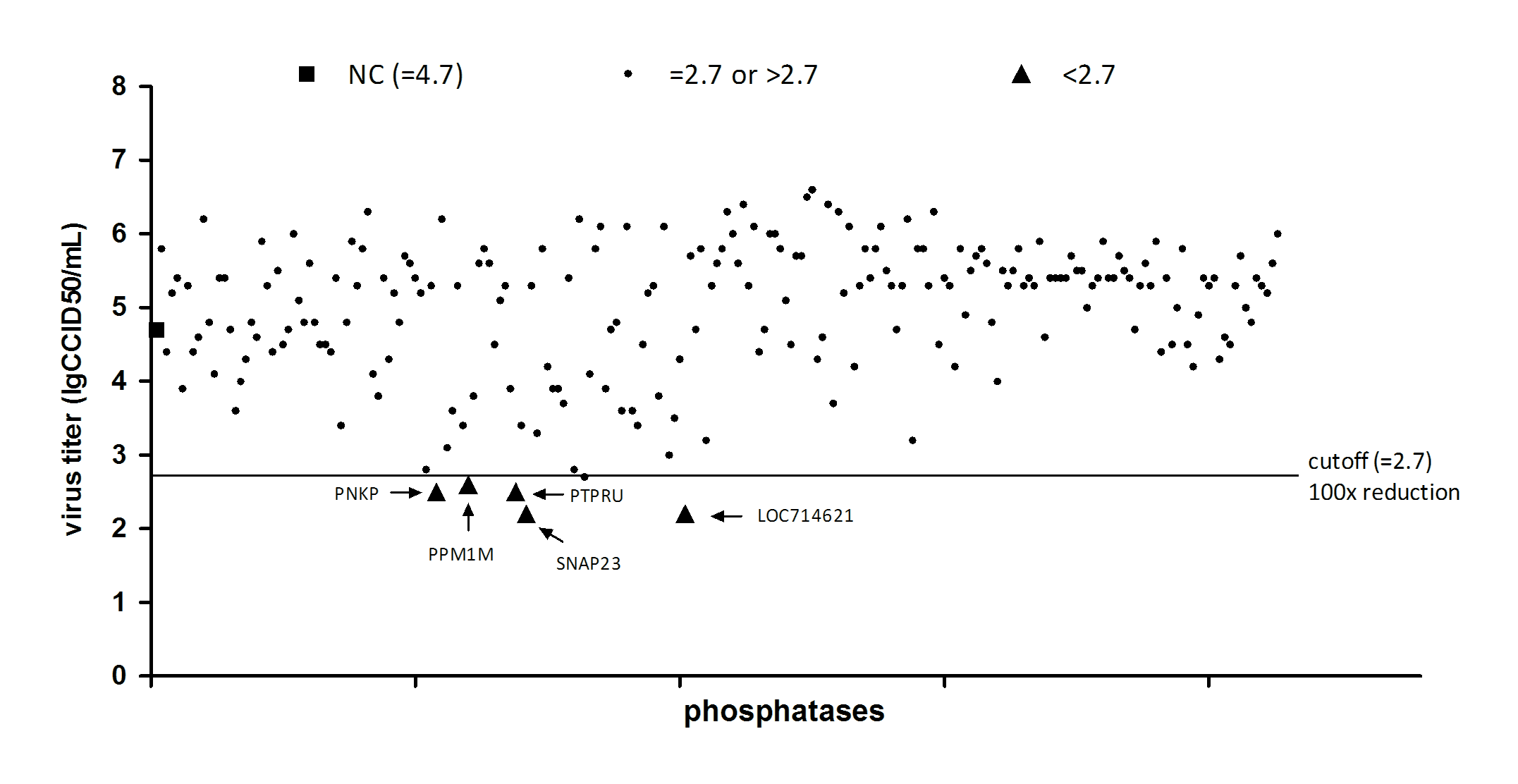

Supplement: Figure S3 — Virus titer for each phosphatase in monkey astrocytes by siRNA screen. Three siRNAs per gene were transfected in astrocytes followed by infection with HSV-1 (at an MOI of 0.01) in three independent experiments. Virus solutions were harvested at 48 h post infection, and the virus titer was determined by the CPE method and analyzed by calculating the lgCCID50/mL. Cut off = 2.7, and it means 100x reduction of HSV-1 replication. NC refers to the negative control. (TIF) [file pone.0079648.s003.tif]
