# Supplementary material for: The Modulation of Phosphatase Expression Impacts the Proliferation Efficiency of HSV-1 in Infected Astrocytes
Source: PLoS One. 2013 Nov 15;8(11):e79648. doi: 10.1371/journal.pone.0079648 (PMC3829861; doi:10.1371/journal.pone.0079648)
Supplement: Table S1 — List of phosphatases in the siRNA library. (DOCX) [file pone.0079648.s004.docx]

**Table S1 List of phosphatases in the siRNA library**

| NO. | Symbol | Accession | Description |
| --- | --- | --- | --- |
| 1 | ACP2 | XM_001109417.2 | PREDICTED: Macaca mulatta acid phosphatase 2, lysosomal |
| 2 | ACP5 | XM_001106957.1 | PREDICTED: Macaca mulatta acid phosphatase 5, tartrate resistant |
| 3 | ACP6 | XM_001099057.2 | PREDICTED: Macaca mulatta acid phosphatase 6, lysophosphatidic |
| 4 | ACPP | XM_001115549.2 | PREDICTED: Macaca mulatta acid phosphatase, prostate |
| 5 | ACPT | XM_001116150.1 | PREDICTED: Macaca mulatta acid phosphatase, testicular |
| 6 | ALPI | XR_014569.2 | PREDICTED: Macaca mulatta intestinal-type alkaline phosphatase-like, miscRNA |
| 7 | ALPL | XM_001109717.2 | PREDICTED: Macaca mulatta alkaline phosphatase, liver/bone/kidney |
| 8 | ALPP | XR_014224.2 | PREDICTED: Macaca mulatta alkaline phosphatase, placental type-like, miscRNA |
| 9 | CDC25A | XM_002802852.1 | PREDICTED: Macaca mulatta cell division cycle 25 homolog A |
| 10 | CDC25C | XM_002804525.1 | PREDICTED: Macaca mulatta cell division cycle 25 homolog C |
| 11 | CDKN1A | NM_001194722.1 | Macaca mulatta cyclin-dependent kinase inhibitor 1A (p21, Cip1) |
| 12 | CDKN1B | XM_001085433.2 | PREDICTED: Macaca mulatta cyclin-dependent kinase inhibitor 1B (p27, Kip1) |
| 13 | CDKN1C | XM_001117302.2 | PREDICTED: Macaca mulatta cyclin-dependent kinase inhibitor 1C (p57, Kip2) |
| 14 | CTDP1 | XM_001088601.2 | PREDICTED: Macaca mulatta CTD (carboxy-terminal domain, RNA polymerase II, polypeptide A) phosphatase, subunit 1 |
| 15 | CTDSP2 | XM_001116527.2 | PREDICTED: Macaca mulatta CTD (carboxy-terminal domain, RNA polymerase II, polypeptide A) small phosphatase 2 |
| 16 | CTDSPL2 | XM_001110308.2 | PREDICTED: Macaca mulatta CTD (carboxy-terminal domain, RNA polymerase II, polypeptide A) small phosphatase like 2 |
| 17 | DUSP10 | XM_001101277. | PREDICTED: Macaca mulatta dual specificity phosphatase 10 |
| 18 | DUSP11 | XM_001105622.2 | PREDICTED: Macaca mulatta dual specificity phosphatase 11 |
| 19 | DUSP13 | XM_002805673.1 | PREDICTED: Macaca mulatta dual specificity phosphatase 13 |
| 20 | DUSP14 | XM_001112007.2 | PREDICTED: Macaca mulatta dual specificity phosphatase 14 |
| 21 | DUSP16 | XM_001084619.2 | PREDICTED: Macaca mulatta dual specificity phosphatase 16 |
| 22 | DUSP18 | XM_001110413.2 | PREDICTED: Macaca mulatta dual specificity phosphatase 18 |
| 23 | DUSP19 | NM_001194218.1\| | Macaca mulatta dual specificity phosphatase 19 |
| 24 | DUSP21 | XM_001088878.2 | PREDICTED: Macaca mulatta dual specificity phosphatase 21 |
| 25 | DUSP22 | XM_001089185.2 | PREDICTED: Macaca mulatta dual specificity phosphatase 22 |
| 26 | DUSP3 | XM_001113754.2 | PREDICTED: Macaca mulatta dual specificity phosphatase 3 |
| 27 | DUSP6 | XM_001101478.2 | PREDICTED: Macaca mulatta dual specificity phosphatase 6 |
| 28 | DUT | XM_001112932.2 | PREDICTED: Macaca mulatta deoxyuridine triphosphatase |
| 29 | ENPP1 | XM_001103359.2 | PREDICTED: Macaca mulatta ectonucleotide pyrophosphatase/phosphodiesterase 1 |
| 30 | ENPP2 | XM_001093656.2 | PREDICTED: Macaca mulatta ectonucleotide pyrophosphatase/phosphodiesterase 2 |
| 31 | ENPP3 | XM_001103528.2 | PREDICTED: Macaca mulatta ectonucleotide pyrophosphatase/phosphodiesterase 3 |
| 32 | ENPP4 | XM_001101698.2 | PREDICTED: Macaca mulatta ectonucleotide pyrophosphatase/phosphodiesterase 4 (putative function) |
| 33 | ENPP5 | XM_001094592.2 | PREDICTED: Macaca mulatta ectonucleotide pyrophosphatase/phosphodiesterase 5 (putative function) |
| 34 | FBP1 | NM_001193652.1 | Macaca mulatta fructose-1,6-bisphosphatase 1 |
| 35 | FBP2 | NM_001194802.1 | Macaca mulatta fructose-1,6-bisphosphatase 2 |
| 36 | FCRL2 | XM_001116902.2 | PREDICTED: Macaca mulatta Fc receptor-like 2 |
| 37 | G6PC3 | XM_001114234.2 | PREDICTED: Macaca mulatta glucose 6 phosphatase, catalytic, 3 |
| 38 | ILKAP | XM_001094705.2 | PREDICTED: Macaca mulatta integrin-linked kinase-associated serine/threonine phosphatase 2C |
| 39 | IMPA1 | XM_001092596.2 | PREDICTED: Macaca mulatta inositol(myo)-1(or 4)-monophosphatase 1 |
| 40 | INPP1 | XM_001095311.1 | PREDICTED: Macaca mulatta inositol polyphosphate-1-phosphatase |
| 41 | INPP4B | XM_002804208.1 | PREDICTED: Macaca mulatta inositol polyphosphate-4-phosphatase, type II, 105kD |
| 42 | INPP5B | XM_002802326.1 | PREDICTED: Macaca mulatta inositol polyphosphate-5-phosphatase, 75kDa |
| 43 | INPP5F | XM_001099262.2 | PREDICTED: Macaca mulatta inositol polyphosphate-5-phosphatase F |
| 44 | INPP5J | XM_001110784.2 | PREDICTED: Macaca mulatta inositol polyphosphate-5-phosphatase J |
| 45 | INPP5K | XM_001117306.2 | PREDICTED: Macaca mulatta inositol polyphosphate-5-phosphatase K |
| 46 | INPPL1 | XM_001114794.2 | PREDICTED: Macaca mulatta inositol polyphosphate phosphatase-like 1 |
| 47 | LHPP | XM_001083060.2 | PREDICTED: Macaca mulatta phospholysine phosphohistidine inorganic pyrophosphate phosphatase |
| 48 | LOC721397 | XM_002799126.1 | PREDICTED: Macaca mulatta low molecular weight phosphotyrosine protein phosphatase-like |
| 49 | LOC694133 | XM_001082837.2 | PREDICTED: Macaca mulatta tyrosine-protein phosphatase non-receptor type 12-like |
| 50 | LOC694255 | XM_001082960.2 | PREDICTED: Macaca mulatta phosphatidylinositol-3,4,5-trisphosphate 3-phosphatase TPTE2-like |
| 51 | LOC694313 | XM_002799888.1 | PREDICTED: Macaca mulatta protein tyrosine phosphatase type IVA 2-like |
| 52 | LOC697073 | XM_001087536.2 | PREDICTED: Macaca mulatta small ubiquitin-related modifier 1-like |
| 53 | LOC698473 | XM_001091142.2 | PREDICTED: Macaca mulatta dual specificity protein phosphatase 7-like |
| 54 | LOC699032 | XM_001087504.2 | PREDICTED: Macaca mulatta receptor-type tyrosine-protein phosphatase H-like |
| 55 | LOC700416 | XM_001088770.2 | PREDICTED: Macaca mulatta phosphatidylinositol-3,4,5-trisphosphate 3-phosphatase TPTE2-like |
| 56 | LOC700574 | XM_001090766.2 | PREDICTED: Macaca mulatta protein phosphatase 1 regulatory subunit 7-like |
| 57 | LOC701032 | XM_001089335.2 | PREDICTED: Macaca mulatta proline-serine-threonine phosphatase-interacting protein 2-like |
| 58 | LOC701233 | XM_001088324.2 | PREDICTED: Macaca mulatta type-1 protein phosphatase inhibitor 4-like |
| 59 | LOC703821 | XM_001092149.2 | PREDICTED: Macaca mulatta protein phosphatase 1 regulatory inhibitor subunit 16B-like |
| 60 | LOC704395 | XM_001092746.2 | PREDICTED: Macaca mulatta phosphatidate phosphatase PPAPDC1B-like |
| 61 | LOC706442 | XM_002798427.1 | PREDICTED: Macaca mulatta dual specificity protein phosphatase 15-like,partial |
| 62 | LOC706539 | XM_001099344.2 | PREDICTED: Macaca mulatta lipid phosphate phosphohydrolase 1-like |
| 63 | LOC708936 | XM_001097826.2 | PREDICTED: Macaca mulatta protein phosphatase inhibitor 2-like |
| 64 | LOC709062 | XM_002798911.1 | PREDICTED: Macaca mulatta glucose-6-phosphatase 2-like |
| 65 | LOC709079 | XM_001097575.2 | PREDICTED: Macaca mulatta protein phosphatase 1 regulatory subunit 14D-like |
| 66 | LOC709364 | XM_002805087.1 | PREDICTED: Macaca mulatta sphingosine-1-phosphate phosphatase 1-like |
| 67 | LOC710326 | XM_001107958.2 | PREDICTED: Macaca mulatta serine/threonine-protein phosphatase 2A catalytic subunit alpha isoform-like |
| 68 | LOC710387 | XM_002808086.1 | PREDICTED: Macaca mulatta serine/threonine-protein phosphatase 2A 65 kDa regulatory subunit A beta isoform-like |
| 69 | LOC711012 | XM_002800438.1 | PREDICTED: Macaca mulatta protein phosphatase 1E-like |
| 70 | LOC711920 | XM_002805296.1 | PREDICTED: Macaca mulatta serine/threonine-protein phosphatase 2A 55 kDa regulatory subunit B alpha isoform-like |
| 71 | LOC712657 | XR_012672.2 | PREDICTED: Macaca mulatta receptor-type tyrosine-protein phosphatase C-like ,miscRNA |
| 72 | LOC713335 | XM_001102648.2 | PREDICTED: Macaca mulatta protein phosphatase 1 regulatory subunit 14B-like |
| 73 | LOC714388 | XM_001105135.2 | PREDICTED: Macaca mulatta lipid phosphate phosphatase-related protein type 2-like |
| 74 | LOC714621 | XM_001104480.2 | PREDICTED: Macaca mulatta myotubularin-related protein 4-like |
| 75 | LOC715302 | XM_001105452.2 | PREDICTED: Macaca mulatta phosphoserine phosphatase-like |
| 76 | LOC715374 | XM_001113078.2 | PREDICTED: Macaca mulatta protein-tyrosine phosphatase-like member B-like |
| 77 | LOC715950 | XM_001106419.2 | PREDICTED: Macaca mulatta protein phosphatase 1 regulatory subunit 3F-like |
| 78 | LOC715993 | XM_001114366.2 | PREDICTED: Macaca mulatta lipid phosphate phosphohydrolase 3-like |
| 79 | LOC716195 | XM_001106788.2 | PREDICTED: Macaca mulatta protein tyrosine phosphatase type IVA 1-like |
| 80 | LOC716496 | XM_001112323.2 | PREDICTED: Macaca mulatta nuclear inhibitor of protein phosphatase 1-like |
| 81 | LOC716874 | XM_001108890.2 | PREDICTED: Macaca mulatta serine/threonine-protein phosphatase 2A activator-like |
| 82 | LOC717257 | XM_001115165.2 | PREDICTED: Macaca mulatta inosine triphosphate pyrophosphatase-like |
| 83 | LOC717432 | XM_001108905.2 | PREDICTED: Macaca mulatta tyrosine-protein phosphatase non-receptor type substrate 1-like |
| 84 | LOC717573 | XR_014379.2 | PREDICTED: Macaca mulatta m-phase inducer phosphatase 2-like, miscRNA |
| 85 | LOC717573 | XR_014379.2 | PREDICTED: Macaca mulatta m-phase inducer phosphatase 2-like, miscRNA |
| 86 | LOC717832 | XM_002799088.1 | PREDICTED: Macaca mulatta phosphatidylinositol-3,4,5-trisphosphate 5-phosphatase 1-like |
| 87 | LOC718442 | XM_001111118.2 | PREDICTED: Macaca mulatta dual specificity protein phosphatase 2-like, partial |
| 88 | LOC719106 | XR_014089.2 | PREDICTED: Macaca mulatta liprin-alpha-3-like, partial miscRNA |
| 89 | LOC719474 | XM_001117253.2 | PREDICTED: Macaca mulatta dual specificity protein phosphatase 23-like |
| 90 | LOC719508 | XM_001113817.2 | PREDICTED: Macaca mulatta type I inositol-1,4,5-trisphosphate 5-phosphatase-like |
| 91 | LOC719523 | XM_001113868.2 | PREDICTED: Macaca mulatta protein phosphatase 1 regulatory subunit 14A-like |
| 92 | LOC720034 | XM_001118147.2 | PREDICTED: Macaca mulatta dual specificity protein phosphatase 12-like |
| 93 | LOC720960 | XM_001116942.2 | PREDICTED: Macaca mulatta dual specificity protein phosphatase 8-like |
| 94 | LOC721004 | XM_001116998.2 | PREDICTED: Macaca mulatta lipid phosphate phosphohydrolase 2-like |
| 95 | LOC721674 | XM_001117869.2 | PREDICTED: Macaca mulatta hypothetical LOC721674 |
| 96 | LOC722026 | XM_001118226.2 | PREDICTED: Macaca mulatta serine/threonine-protein phosphatase 2A 56 kDa regulatory subunit beta isoform-like |
| 97 | LOC722572 | XM_001118701.2 | PREDICTED: Macaca mulatta dolichyldiphosphatase 1-like |
| 98 | MINPP1 | XM_001101346.2 | PREDICTED: Macaca mulatta multiple inositol polyphosphate histidine phosphatase, 1 |
| 99 | MTM1 | XM_002806430.1 | PREDICTED: Macaca mulatta myotubularin |
| 100 | MTMR12 | XM_001089010.2 | PREDICTED: Macaca mulatta myotubularin related protein 12 |
| 101 | MTMR2 | XM_001092318.2 | PREDICTED: Macaca mulatta myotubularin related protein 2 |
| 102 | MTMR3 | XM_001107250.2 | PREDICTED: Macaca mulatta myotubularin related protein 3 |
| 103 | MTMR6 | XM_001092084.1 | PREDICTED: Macaca mulatta myotubularin related protein 6 |
| 104 | MTMR8 | XM_001097351.1 | PREDICTED: Macaca mulatta myotubularin related protein 8 |
| 105 | MTMR9 | XM_001088743.2 | PREDICTED: Macaca mulatta myotubularin related protein 9 |
| 106 | PDP1 | XM_002805442.1 | PREDICTED: Macaca mulatta pyruvate dehyrogenase phosphatase catalytic subunit 1 |
| 107 | PDP2 | XM_002802527.1 | PREDICTED: Macaca mulatta pyruvate dehyrogenase phosphatase catalytic subunit 2 |
| 108 | PDPR | XM_001108170.2 | PREDICTED: Macaca mulatta pyruvate dehydrogenase phosphatase regulatory subunit, mitochondrial-like |
| 109 | PHLPP1 | XM_001094216.2 | PREDICTED: Macaca mulatta PH domain and leucine rich repeat protein phosphatase 1 |
| 110 | PHLPP2 | XM_001105985.2 | PREDICTED: Macaca mulatta PH domain and leucine rich repeat protein phosphatase 2 |
| 111 | PHOSPHO1 | XM_001092436.2 | PREDICTED: Macaca mulatta phosphatase, orphan 1 |
| 112 | PHOSPHO2 | NM_001194769.1 | Macaca mulatta phosphatase, orphan 2 |
| 113 | PMPCA | NM_001194013.1 | Macaca mulatta peptidase (mitochondrial processing) alpha, nuclear gene encoding mitochondrial protein |
| 114 | PNKP | XM_001115581.1 | PREDICTED: Macaca mulatta polynucleotide kinase 3'-phosphatase |
| 115 | PPAPDC1A | NM_001266703.1 | Macaca mulatta phosphatidic acid phosphatase type 2 domain containing 1A |
| 116 | PPAPDC2 | NM_001198707.1 | Macaca mulatta phosphatidic acid phosphatase type 2 domain containing 2 |
| 117 | PPEF1 | XM_001083653.2 | PREDICTED: Macaca mulatta protein phosphatase, EF-hand calcium binding domain 1 |
| 118 | PPEF2 | XM_001100042.2 | PREDICTED: Macaca mulatta protein phosphatase, EF-hand calcium binding domain 2 |
| 119 | PPFIA1 | XM_002799457.1 | PREDICTED: Macaca mulatta protein tyrosine phosphatase, receptor type, f polypeptide (PTPRF), interacting protein (liprin), alpha 1 |
| 120 | PPFIA2 | XM_001088353.2 | PREDICTED: Macaca mulatta protein tyrosine phosphatase, receptor type, f polypeptide (PTPRF), interacting protein (liprin), alpha 2 |
| 121 | PPFIA4 | XM_001104868.2 | PREDICTED: Macaca mulatta protein tyrosine phosphatase, receptor type, f polypeptide (PTPRF), interacting protein (liprin), alpha 4 |
| 122 | PPM1A | XM_002805056.1 | PREDICTED: Macaca mulatta protein phosphatase, Mg2+/Mn2+ dependent, 1A |
| 123 | PPM1B | XM_001111662.2 | PREDICTED: Macaca mulatta protein phosphatase, Mg2+/Mn2+ dependent, 1B |
| 124 | PPM1D | XM_001110891.2 | PREDICTED: Macaca mulatta protein phosphatase, Mg2+/Mn2+ dependent, 1D |
| 125 | PPM1F | XM_001089477.2 | PREDICTED: Macaca mulatta protein phosphatase, Mg2+/Mn2+ dependent, 1F |
| 126 | PPM1G | XM_001096179.2 | PREDICTED: Macaca mulatta protein phosphatase, Mg2+/Mn2+ dependent, 1G |
| 127 | PPM1H | XM_001116776.2 | PREDICTED: Macaca mulatta protein phosphatase, Mg2+/Mn2+ dependent, 1H |
| 128 | PPM1J | XM_001096598.2 | PREDICTED: Macaca mulatta protein phosphatase, Mg2+/Mn2+ dependent, 1J |
| 129 | PPM1K | XM_001100402.1 | PREDICTED: Macaca mulatta protein phosphatase, Mg2+/Mn2+ dependent, 1K |
| 130 | PPM1L | XM_001096769.2 | PREDICTED: Macaca mulatta protein phosphatase 1L-like |
| 131 | PPM1M | NM_001193781.1 | Macaca mulatta protein phosphatase, Mg2+/Mn2+ dependent, 1M |
| 132 | PPME1 | XM_001115651.2 | PREDICTED: Macaca mulatta protein phosphatase methylesterase 1 |
| 133 | PPP1CA | XM_002799468.1 | PREDICTED: Macaca mulatta protein phosphatase 1, catalytic subunit, alpha isozyme |
| 134 | PPP1CB | XM_001102036.2 | PREDICTED: Macaca mulatta protein phosphatase 1, catalytic subunit, beta isozyme |
| 135 | PPP1CC | XM_001108424.2 | PREDICTED: Macaca mulatta protein phosphatase 1, catalytic subunit, gamma isozyme |
| 136 | PPP1R10 | NM_001114944.1 | Protein phosphatase 1, regulatory (inhibitor) subunit 10 |
| 137 | PPP1R11 | NM_001047136.1 | Protein phosphatase 1, regulatory (inhibitor) subunit 11 |
| 138 | PPP1R12A | XM_001086127.2 | PREDICTED: Macaca mulatta protein phosphatase 1, regulatory (inhibitor) subunit 12A |
| 139 | PPP1R12B | XM_001106168.2 | PREDICTED: Macaca mulatta protein phosphatase 1, regulatory (inhibitor) subunit 12B |
| 140 | PPP1R12C | XM_001087036.2 | PREDICTED: Macaca mulatta protein phosphatase 1, regulatory (inhibitor) subunit 12C |
| 141 | PPP1R13B | XM_001088783.2 | PREDICTED: Macaca mulatta protein phosphatase 1, regulatory (inhibitor) subunit 13B |
| 142 | PPP1R15A | XM_001114376.2 | PREDICTED: Macaca mulatta protein phosphatase 1, regulatory (inhibitor) subunit 15A |
| 143 | PPP1R15B | XM_001097695.2 | PREDICTED: Macaca mulatta protein phosphatase 1, regulatory (inhibitor) subunit 15B |
| 144 | PPP1R1A | XM_001110247.2 | PREDICTED: Macaca mulatta protein phosphatase 1 |
| 145 | PPP1R1B | XM_001089386.2 | PREDICTED: Macaca mulatta protein phosphatase 1, regulatory (inhibitor) subunit 1B |
| 146 | PPP1R1C | XM_001101383.2 | PREDICTED: Macaca mulatta protein phosphatase 1, regulatory (inhibitor) subunit 1C |
| 147 | PPP1R3A | XM_001100958.2 | PREDICTED: Macaca mulatta protein phosphatase 1, regulatory (inhibitor) subunit 3A |
| 148 | PPP1R3B | XM_001090708.1 | PREDICTED: Macaca mulatta protein phosphatase 1, regulatory (inhibitor) subunit 3B |
| 149 | PPP1R3C | XM_001088532.1 | PREDICTED: Macaca mulatta protein phosphatase 1, regulatory (inhibitor) subunit 3C |
| 150 | PPP1R3D | XM_001090753.2 | PREDICTED: Macaca mulatta protein phosphatase 1, regulatory (inhibitor) subunit 3D |
| 151 | PPP2CB | XM_001084560.2 | PREDICTED: Macaca mulatta protein phosphatase 2, catalytic subunit, beta isozyme |
| 152 | PPP2R1A | XM_001116604.2 | PREDICTED: Macaca mulatta protein phosphatase 2, regulatory subunit A, alpha |
| 153 | PPP2R2B | XM_001095405.2 | PREDICTED: Macaca mulatta protein phosphatase 2, regulatory subunit B, beta |
| 154 | PPP2R2C | XM_001092928.2 | PREDICTED: Macaca mulatta protein phosphatase 2, regulatory subunit B, gamma |
| 155 | PPP2R2D | XM_001091312.2 | PREDICTED: Macaca mulatta serine/threonine-protein phosphatase 2A 55 kDa regulatory subunit B delta isoform-like |
| 156 | PPP2R3A | XM_001115154.2 | PREDICTED: Macaca mulatta protein phosphatase 2 (formerly 2A), regulatory subunit B'', alpha |
| 157 | PPP2R5A | XM_001106979.2 | PREDICTED: Macaca mulatta protein phosphatase 2, regulatory subunit B', alpha |
| 158 | PPP2R5C | XM_002805213.1 | PREDICTED: Macaca mulatta protein phosphatase 2, regulatory subunit B', gamma |
| 159 | PPP2R5D | XM_002803743.1 | PREDICTED: Macaca mulatta protein phosphatase 2, regulatory subunit B', delta |
| 160 | PPP2R5E | XM_002805064.1 | PREDICTED: Macaca mulatta protein phosphatase 2, regulatory subunit B', epsilon isoform |
| 161 | PPP3CA | XM_001108659.2 | PREDICTED: Macaca mulatta protein phosphatase 3, catalytic subunit, alpha isozyme |
| 162 | PPP3CB | XM_001101616.2 | PREDICTED: Macaca mulatta protein phosphatase 3, catalytic subunit, beta isozyme |
| 163 | PPP3CC | XM_001103623.2 | PREDICTED: Macaca mulatta protein phosphatase 3, catalytic subunit, gamma isozyme |
| 164 | PPP3R2 | NM_001195420.1 | Macaca mulatta protein phosphatase 3, regulatory subunit B, beta |
| 165 | PPP4C | XM_001108500.2 | PREDICTED: Macaca mulatta protein phosphatase 4, catalytic subunit |
| 166 | PPP4R1 | XM_001098097.2 | PREDICTED: Macaca mulatta protein phosphatase 4, regulatory subunit 1 |
| 167 | PPP4R2 | XM_002802736.1 | PREDICTED: Macaca mulatta protein phosphatase 4, regulatory subunit 2 |
| 168 | PPP5C | XM_001111749.2 | PREDICTED: Macaca mulatta protein phosphatase 5, catalytic subunit |
| 169 | PPP6C | XM_001099210.2 | PREDICTED: Macaca mulatta protein phosphatase 6, catalytic subunit |
| 170 | PPTC7 | XM_001107446.2 | PREDICTED: Macaca mulatta protein phosphatase PTC7 homolog |
| 171 | PSTPIP2 | XM_001105723.2 | PREDICTED: Macaca mulatta proline-serine-threonine phosphatase-interacting protein 1-like |
| 172 | PTEN | XM_001102542.2 | PREDICTED: Macaca mulatta phosphatase and tensin homolog |
| 173 | PTP4A3 | NM_001193694.1 | Macaca mulatta protein tyrosine phosphatase type IVA, member 3 |
| 174 | PTPDC1 | XM_002800194.1 | PREDICTED: Macaca mulatta protein tyrosine phosphatase domain containing 1 |
| 175 | PTPLA | NM_001193912.1 | Macaca mulatta protein tyrosine phosphatase-like (proline instead of catalytic arginine), member A |
| 176 | PTPLAD1 | XM_002804825.1 | PREDICTED: Macaca mulatta protein tyrosine phosphatase-like A domain containing 1 |
| 177 | PTPLAD2 | NM_001194139.1 | Macaca mulatta protein tyrosine phosphatase-like A domain containing 2 |
| 178 | PTPMT1 | XM_001105550.2 | PREDICTED: Macaca mulatta protein tyrosine phosphatase, mitochondrial 1 |
| 179 | PTPN1 | XM_001096053.2 | PREDICTED: Macaca mulatta protein tyrosine phosphatase, non-receptor type 1 |
| 180 | PTPN11 | XM_001110117.2 | PREDICTED: Macaca mulatta protein tyrosine phosphatase, non-receptor type 11 |
| 181 | PTPN13 | XM_002808414.1 | PREDICTED: Macaca mulatta tyrosine-protein phosphatase non-receptor type 13-like |
| 182 | PTPN14 | XM_001106232.2 | PREDICTED: Macaca mulatta protein tyrosine phosphatase, non-receptor type 14 |
| 183 | PTPN18 | XM_001093333.1 | PREDICTED: Macaca mulatta protein tyrosine phosphatase, non-receptor type 18 (brain-derived) |
| 184 | PTPN2 | XM_001118072.2 | PREDICTED: Macaca mulatta protein tyrosine phosphatase, non-receptor type 2 |
| 185 | PTPN20A | XM_001109236.2 | PREDICTED: Macaca mulatta protein tyrosine phosphatase, non-receptor type 20A |
| 186 | PTPN21 | XM_001083418.2 | PREDICTED: Macaca mulatta protein tyrosine phosphatase, non-receptor type 21 |
| 187 | PTPN22 | XM_001110200.2 | PREDICTED: Macaca mulatta protein tyrosine phosphatase, non-receptor type 22 (lymphoid) |
| 188 | PTPN23 | XM_001100428.2 | PREDICTED: Macaca mulatta protein tyrosine phosphatase, non-receptor type 23 |
| 189 | PTPN3 | XM_001107076.2 | PREDICTED: Macaca mulatta protein tyrosine phosphatase, non-receptor type 3 |
| 190 | PTPN4 | XM_001084627.2 | PREDICTED: Macaca mulatta protein tyrosine phosphatase, non-receptor type 4 |
| 191 | PTPN5 | XM_002799610.1 | PREDICTED: Macaca mulatta protein tyrosine phosphatase, non-receptor type 5 (striatum-enriched) |
| 192 | PTPN6 | XM_001110915.2 | PREDICTED: Macaca mulatta protein tyrosine phosphatase, non-receptor type 6 |
| 193 | PTPN7 | XM_001106613.2 | PREDICTED: Macaca mulatta protein tyrosine phosphatase, non-receptor type 7 |
| 194 | PTPN9 | XM_002804881.1 | PREDICTED: Macaca mulatta protein tyrosine phosphatase, non-receptor type 9 |
| 195 | PTPRA | XM_002798218.1 | PREDICTED: Macaca mulatta protein tyrosine phosphatase, receptor type, A |
| 196 | PTPRB | XM_001117455.2 | PREDICTED: Macaca mulatta protein tyrosine phosphatase, receptor type, B |
| 197 | PTPRD | XM_001112144.2 | PREDICTED: Macaca mulatta protein tyrosine phosphatase, receptor type, D |
| 198 | PTPRE | XM_002805863.1 | PREDICTED: Macaca mulatta receptor-type tyrosine-protein phosphatase epsilon-like |
| 199 | PTPRF | XM_001095521.2 | PREDICTED: Macaca mulatta protein tyrosine phosphatase, receptor type, F |
| 200 | PTPRG | XM_002802749.1 | PREDICTED: Macaca mulatta protein tyrosine phosphatase, receptor type, G |
| 201 | PTPRJ | XM_002799572.1 | PREDICTED: Macaca mulatta protein tyrosine phosphatase, receptor type, J |
| 202 | PTPRK | XM_002803929.1 | PREDICTED: Macaca mulatta protein tyrosine phosphatase, receptor type, K |
| 203 | PTPRN | XM_002799079.1 | PREDICTED: Macaca mulatta protein tyrosine phosphatase, receptor type, N |
| 204 | PTPRO | XM_002798510.1 | PREDICTED: Macaca mulatta protein tyrosine phosphatase, receptor type, O |
| 205 | PTPRR | NM_001193766.1 | Macaca mulatta protein tyrosine phosphatase, receptor type, R |
| 206 | PTPRS | XM_001083499.2 | PREDICTED: Macaca mulatta protein tyrosine phosphatase, receptor type, S |
| 207 | PTPRU | XM_001115805.2 | PREDICTED: Macaca mulatta protein tyrosine phosphatase, receptor type, U |
| 208 | RNGTT | XM_001090617.2 | PREDICTED: Macaca mulatta RNA guanylyltransferase and 5'-phosphatase |
| 209 | SBF1 | XR_014073.2 | PREDICTED: Macaca mulatta myotubularin-related protein 5-like, miscRNA |
| 210 | SLC37A4 | NM_001194790.1 | Macaca mulatta solute carrier family 37 (glucose-6-phosphate transporter), member 4 |
| 211 | SNAP23 | XM_002804738.1 | PREDICTED: Macaca mulatta synaptosomal-associated protein 23-like |
| 212 | TENC1 | XM_001102202.2 | PREDICTED: Macaca mulatta tensin like C1 domain containing phosphatase (tensin 2) |
