# Supplementary material for: The Modulation of Phosphatase Expression Impacts the Proliferation Efficiency of HSV-1 in Infected Astrocytes
Source: PLoS One. 2013 Nov 15;8(11):e79648. doi: 10.1371/journal.pone.0079648 (PMC3829861; doi:10.1371/journal.pone.0079648)
Supplement: Table S2 — Normalized virus titers for RNAi screening. (DOCX) [file pone.0079648.s005.docx]

**Table S2 Normalized virus titers for RNAi screening**

| NAME | MEAN  (lgCCID50/ml) | SD | Fold of virus titer  ---Target (CCID50/ml) / NC(CCID50/ml) |
| --- | --- | --- | --- |
| NC | 4.7 | 0.9 | 1 |
| ACP2 | 5.8 | 0.7 | 12.589 |
| ACP5 | 4.4 | 1.5 | 0.501 |
| ACP6 | 5.2 | 0.7 | 3.162 |
| ACPP | 5.4 | 1.3 | 5.012 |
| ACPT | 3.9 | 1.4 | 0.158 |
| ALPL | 5.3 | 0.4 | 3.981 |
| CDC25A | 4.4 | 1.5 | 0.501 |
| CDC25C | 4.6 | 1.0 | 0.794 |
| CDKN1A | 6.2 | 0.3 | 31.623 |
| CDKN1B | 4.8 | 1.3 | 1.259 |
| CDKN1C | 4.1 | 1.6 | 0.251 |
| CTDP1 | 5.4 | 0.4 | 5.012 |
| CTDSP2 | 5.4 | 0.8 | 5.012 |
| DUSP3 | 4.7 | 1.2 | 1 |
| DUSP6 | 3.6 | 0.4 | 0.079 |
| DUSP10 | 4.0 | 0.9 | 0.2 |
| CTDSPL2 | 4.3 | 0.3 | 0.398 |
| DUSP11 | 4.8 | 1.5 | 1.259 |
| DUSP13 | 4.6 | 1.8 | 0.794 |
| DUSP14 | 5.9 | 0.4 | 15.849 |
| DUSP16 | 5.3 | 2.3 | 3.981 |
| DUSP18 | 4.4 | 1.2 | 0.501 |
| DUSP19 | 5.5 | 0.9 | 6.31 |
| DUSP21 | 4.5 | 0.9 | 0.631 |
| DUSP22 | 4.7 | 0.6 | 1 |
| DUT | 6.0 | 0.4 | 19.953 |
| ENPP1 | 5.1 | 0.8 | 2.512 |
| ENPP2 | 4.8 | 1.4 | 1.259 |
| ENPP3 | 5.6 | 0.2 | 7.943 |
| ENPP4 | 4.8 | 1.1 | 1.259 |
| ENPP5 | 4.5 | 2.4 | 0.631 |
| FBP1 | 4.5 | 0.9 | 0.631 |
| FBP2 | 4.4 | 1.8 | 0.501 |
| FCRL2 | 5.4 | 1.2 | 5.012 |
| G6PC3 | 3.4 | 0.5 | 0.05 |
| SLC37A4 | 4.8 | 0.0 | 1.259 |
| IMPA1 | 5.9 | 0.1 | 15.849 |
| INPP5B | 5.3 | 0.0 | 3.981 |
| INPP5F | 5.8 | 0.9 | 12.589 |
| INPP5J | 6.3 | 0.4 | 39.811 |
| INPP5K | 4.1 | 0.5 | 0.251 |
| INPPL1 | 3.8 | 1.8 | 0.126 |
| LHPP | 5.4 | 0.4 | 5.012 |
| LOC721397 | 4.3 | 0.8 | 0.398 |
| INPP4B | 5.2 | 1.1 | 3.162 |
| LOC694255 | 4.8 | 1.0 | 1.259 |
| LOC698473 | 5.7 | 0.4 | 10 |
| LOC700416 | 5.6 | 0.6 | 7.943 |
| LOC701233 | 5.4 | 1.0 | 5.012 |
| LOC704395 | 5.2 | 1.1 | 3.162 |
| PHOSPHO1 | 2.8 | 1.1 | 0.013 |
| PMPCA | 5.3 | 1.0 | 3.981 |
| **PNKP** | **2.5** | **0.9** | **0.006** |
| PPAPDC1A | 6.2 | 0.4 | 31.623 |
| PPAPDC2 | 3.1 | 0.9 | 0.025 |
| PPFIA1 | 3.6 | 1.6 | 0.079 |
| PPFIA2 | 5.3 | 0.4 | 3.981 |
| PPFIA4 | 3.4 | 0.9 | 0.05 |
| **PPM1M** | **2.6** | **0.6** | **0.008** |
| PPME1 | 3.8 | 0.6 | 0.126 |
| PPP1R1C | 5.6 | 0.6 | 7.943 |
| PPTC7 | 5.8 | 0.1 | 12.589 |
| PTEN | 5.6 | 0.1 | 7.943 |
| PTPMT1 | 4.5 | 0.5 | 0.631 |
| PTPN20A | 5.1 | 1.4 | 2.512 |
| INPP1 | 5.3 | 0.7 | 3.981 |
| PTPRR | 3.9 | 1.3 | 0.158 |
| **PTPRU** | **2.5** | **0.8** | **0.006** |
| RNGTT | 3.4 | 1.0 | 0.05 |
| **SNAP23** | **2.2** | **0.1** | **0.003** |
| TENC1 | 5.3 | 0.9 | 3.981 |
| LOC706442 | 3.3 | 1.2 | 0.04 |
| LOC717573 | 5.8 | 0.5 | 12.589 |
| PHOSPHO2 | 4.2 | 1.9 | 0.316 |
| LOC694133 | 3.9 | 1.2 | 0.158 |
| PPEF1 | 3.9 | 2.5 | 0.158 |
| PTPLA | 3.7 | 1.4 | 0.1 |
| ILKAP | 5.4 | 0.1 | 5.012 |
| PPEF2 | 2.8 | 1.1 | 0.013 |
| LOC694313 | 6.2 | 0.4 | 31.623 |
| PTP4A3 | 2.7 | 1.0 | 0.01 |
| LOC699032 | 4.1 | 0.6 | 0.251 |
| LOC700574 | 5.8 | 0.8 | 12.589 |
| LOC701032 | 6.1 | 0.3 | 25.119 |
| LOC703821 | 3.9 | 1.0 | 0.158 |
| LOC708936 | 4.7 | 1.2 | 1 |
| LOC709079 | 4.8 | 0.4 | 1.259 |
| LOC710326 | 3.6 | 1.9 | 0.079 |
| LOC710387 | 6.1 | 0.4 | 25.119 |
| LOC711012 | 3.6 | 1.3 | 0.079 |
| LOC711920 | 3.4 | 0.9 | 0.05 |
| LOC712657 | 4.5 | 1.1 | 0.631 |
| LOC715374 | 5.2 | 0.9 | 3.162 |
| LOC715950 | 5.3 | 0.7 | 3.981 |
| LOC716496 | 3.8 | 1.3 | 0.126 |
| LOC706539 | 6.1 | 0.5 | 25.119 |
| LOC709062 | 3.0 | 1.5 | 0.02 |
| LOC709364 | 3.5 | 1.1 | 0.063 |
| LOC714388 | 4.3 | 1.3 | 0.398 |
| **LOC714621** | **2.2** | **0.7** | **0.003** |
| LOC715302 | 5.7 | 0.6 | 10 |
| LOC715993 | 4.7 | 0.8 | 1 |
| LOC717257 | 5.8 | 0.6 | 12.589 |
| LOC717832 | 3.2 | 1.8 | 0.032 |
| LOC719474 | 5.3 | 0.6 | 3.981 |
| LOC719508 | 5.6 | 0.6 | 7.943 |
| LOC720034 | 5.8 | 0.7 | 12.589 |
| LOC720960 | 6.3 | 0.7 | 39.811 |
| LOC721004 | 6.0 | 0.4 | 19.953 |
| LOC721674 | 5.6 | 0.4 | 7.943 |
| LOC722572 | 6.4 | 0.2 | 50.119 |
| MINPP1 | 5.3 | 1.2 | 3.981 |
| MTM1 | 6.1 | 0.4 | 25.119 |
| MTMR2 | 4.4 | 2.5 | 0.501 |
| MTMR3 | 4.7 | 1.6 | 1 |
| MTMR6 | 6.0 | 0.3 | 19.953 |
| MTMR9 | 6.0 | 0.4 | 19.953 |
| MTMR12 | 5.8 | 0.4 | 12.589 |
| PDP1 | 5.1 | 1.8 | 2.512 |
| PDP2 | 4.5 | 2.4 | 0.631 |
| PDPR | 5.7 | 0.1 | 10 |
| LOC716874 | 5.7 | 0.6 | 10 |
| LOC717432 | 6.5 | 0.3 | 63.096 |
| LOC719523 | 6.6 | 0.1 | 79.433 |
| LOC722026 | 4.3 | 1.2 | 0.398 |
| PHLPP1 | 4.6 | 2.5 | 0.794 |
| PHLPP2 | 6.4 | 0.4 | 50.119 |
| PPM1B | 3.7 | 2.0 | 0.1 |
| PPM1D | 6.3 | 0.6 | 39.811 |
| PPM1F | 5.2 | 0.6 | 3.162 |
| PPM1G | 6.1 | 0.4 | 25.119 |
| PPM1H | 4.2 | 2.1 | 0.316 |
| PPM1J | 5.3 | 0.5 | 3.981 |
| PPM1K | 5.8 | 0.9 | 12.589 |
| PPM1L | 5.4 | 0.5 | 5.012 |
| PPP1CA | 5.8 | 1.1 | 12.589 |
| PPP1CB | 6.1 | 0.5 | 25.119 |
| PPP1CC | 5.5 | 1.3 | 6.31 |
| PPP1R10 | 5.3 | 1.6 | 3.981 |
| PPP1R12A | 4.7 | 2.1 | 1 |
| PPP1R12B | 5.3 | 0.7 | 3.981 |
| PPP1R13B | 6.2 | 1.0 | 31.623 |
| PPP1R15A | 3.2 | 2.2 | 0.032 |
| PPP1R1A | 5.8 | 0.4 | 12.589 |
| PPP1R3A | 5.8 | 0.9 | 12.589 |
| PPP1R3D | 5.3 | 1.1 | 3.981 |
| PPP1R15B | 6.3 | 0.4 | 39.811 |
| PPP1R1B | 4.5 | 2.2 | 0.631 |
| ALPI | 5.4 | 0.9 | 5.012 |
| ALPP | 5.3 | 0.7 | 3.981 |
| LOC697073 | 4.2 | 2.1 | 0.316 |
| LOC713335 | 5.8 | 0.3 | 12.589 |
| LOC716195 | 4.9 | 1.5 | 1.585 |
| PPP1R11 | 5.5 | 0.7 | 6.31 |
| PPP1R12C | 5.7 | 0.1 | 10 |
| LOC718442 | 5.8 | 0.4 | 12.589 |
| LOC719106 | 5.6 | 0.5 | 7.943 |
| SBF1 | 4.8 | 1.2 | 1.259 |
| MTMR8 | 4.0 | 2.8 | 0.2 |
| PTPRS | 5.5 | 0.3 | 6.31 |
| PPMIA | 5.3 | 0.4 | 3.981 |
| PPP1R3B | 5.5 | 0.7 | 6.31 |
| PPP1R3C | 5.8 | 0.1 | 12.589 |
| PPP2CB | 5.3 | 0.7 | 3.981 |
| PPP2R1A | 5.4 | 0.3 | 5.012 |
| PPP2R2B | 5.3 | 0.8 | 3.981 |
| PPP2R2C | 5.9 | 0.1 | 15.849 |
| PPP2R2D | 4.6 | 1.8 | 0.794 |
| PPP2R3A | 5.4 | 0.5 | 5.012 |
| PPP2R5A | 5.4 | 0.4 | 5.012 |
| PPP2R5C | 5.4 | 0.4 | 5.012 |
| PPP2R5D | 5.4 | 0.6 | 5.012 |
| PPP2R5E | 5.7 | 0.6 | 10 |
| PPP3CA | 5.5 | 0.8 | 6.31 |
| PPP3CB | 5.5 | 0.3 | 6.31 |
| PPP3CC | 5.0 | 1.3 | 1.995 |
| PPP3R2 | 5.3 | 0.1 | 3.981 |
| PPP4C | 5.4 | 0.2 | 5.012 |
| PPP4R1 | 5.9 | 0.6 | 15.849 |
| PPP4R2 | 5.4 | 0.1 | 5.012 |
| PPP5C | 5.4 | 0.4 | 5.012 |
| PPP6C | 5.7 | 0.6 | 10 |
| PSTPIP2 | 5.5 | 0.6 | 6.31 |
| PTPDC1 | 5.4 | 0.7 | 5.012 |
| PTPLAD1 | 4.7 | 1.9 | 1 |
| PTPLAD2 | 5.3 | 0.1 | 3.981 |
| PTPN1 | 5.6 | 0.3 | 7.943 |
| PTPN11 | 5.3 | 0.5 | 3.981 |
| PTPN13 | 5.9 | 0.4 | 15.849 |
| PTPN14 | 4.4 | 2.3 | 0.501 |
| PTPN18 | 5.4 | 0.5 | 5.012 |
| PTPN2 | 4.5 | 1.3 | 0.631 |
| PTPN20A | 5.0 | 0.2 | 1.995 |
| PTPN21 | 5.8 | 0.9 | 12.589 |
| PTPN22 | 4.5 | 1.5 | 0.631 |
| PTPN23 | 4.2 | 1.5 | 0.316 |
| PTPN3 | 4.9 | 0.7 | 1.585 |
| PTPN4 | 5.4 | 0.1 | 5.012 |
| PTPN5 | 5.3 | 0.7 | 3.981 |
| PTPN6 | 5.4 | 0.1 | 5.012 |
| PTPN7 | 4.3 | 1.3 | 0.398 |
| PTPN9 | 4.6 | 1.0 | 0.794 |
| PTPRA | 4.5 | 1.6 | 0.631 |
| PTPRB | 5.3 | 0.4 | 3.981 |
| PTPRD | 5.7 | 0.5 | 10 |
| PTPRE | 5.0 | 0.3 | 1.995 |
| PTPRF | 4.8 | 1.5 | 1.259 |
| PTPRG | 5.4 | 0.4 | 5.012 |
| PTPRJ | 5.3 | 0.3 | 3.981 |
| PTPRK | 5.2 | 0.3 | 3.162 |
| PTPRN | 5.6 | 0.3 | 7.943 |
| PTPRO | 6.0 | 0.9 | 19.953 |
| Cutoff | 2.7 |  | 0.01  (100x reduction) |
